# Generation and Behavior Characterization of CaMKIIβ Knockout Mice

**DOI:** 10.1371/journal.pone.0105191

**Published:** 2014-08-15

**Authors:** Adam D. Bachstetter, Scott J. Webster, Tao Tu, Danielle S. Goulding, Jacques Haiech, D. Martin Watterson, Linda J. Van Eldik

**Affiliations:** 1 Sanders-Brown Center on Aging, University of Kentucky, Lexington, Kentucky, United States of America; 2 Department of Anatomy and Neurobiology, University of Kentucky, Lexington, Kentucky, United States of America; 3 Department of Molecular Pharmacology and Biological Chemistry, Northwestern University Feinberg School of Medicine, Chicago, Illinois, United States of America; 4 Laboratoire d'Innovation Thérapeutique, University of Strasbourg, Strasbourg, France; Alexander Fleming Biomedical Sciences Research Center, Greece

## Abstract

The calcium/calmodulin-dependent protein kinase II (CaMKII) is abundant in the brain, where it makes important contributions to synaptic organization and homeostasis, including playing an essential role in synaptic plasticity and memory. Four genes encode isoforms of CaMKII (α, β, δ, γ), with CaMKIIα and CaMKIIβ highly expressed in the brain. Decades of molecular and cellular research, as well as the use of a large number of CaMKIIα mutant mouse lines, have provided insight into the pivotal roles of CaMKIIα in brain plasticity and cognition. However, less is known about the CaMKIIβ isoform. We report the development and extensive behavioral and phenotypic characterization of a CaMKIIβ knockout (KO) mouse. The CaMKIIβ KO mouse was found to be smaller at weaning, with an altered body mass composition. The CaMKIIβ KO mouse showed ataxia, impaired forelimb grip strength, and deficits in the rotorod, balance beam and running wheel tasks. Interestingly, the CaMKIIβ KO mouse exhibited reduced anxiety in the elevated plus maze and open field tests. The CaMKIIβ KO mouse also showed cognitive impairment in the novel object recognition task. Our results provide a comprehensive behavioral characterization of mice deficient in the β isoform of CaMKII. The neurologic phenotypes and the construction of the genotype suggest the utility of this KO mouse strain for future studies of CaMKIIβ in brain structure, function and development.

## Introduction

The calcium/calmodulin-dependent protein kinase II (CaMKII) family is key to transducing calcium signals into biological responses, especially in the central nervous system (CNS). There are four isoforms of CaMKII (αβγδ) in the mammalian genome, which are encoded by separate genes. The CaMKIIαand CaMKIIβisoforms are expressed predominantly by neurons and together account for approximately 1–2% of the total protein in the CNS (for reviews see: [1], [2]). The γandδ isoforms are expressed at much lower levels in the brain and primarily by astrocytes [3].

CaMKIIα and CaMKIIβ are both expressed at high levels in the hippocampus, where the role of the kinase in hippocampal long-term potentiation (LTP) is well described [4], [5]). Interestingly, the α and β isoforms of CaMKII have been reported to have independent but essential functions in maintaining LTP [1], [2], [5]. CaMKIIβ may specifically affect synaptic function by altering the size and shape of the dendritic spines [6]–[8], or by localizing CaMKIIα to the dendritic spines [9].

Regional differences exist in the expression of the CaMKIIα and CaMKIIβ isoforms. For example, CaMKIIβ is the dominant isoform in the cerebellum, and a role for CaMKIIβin cerebellar physiology has been described [10]. While the β isoform of CaMKII clearly has important roles in synaptic organization and function, nearly all the reported functions for CaMKII are focused on CaMKIIα [4], [5]. The regional differences in expression of the CaMKIIβ isoform compared to CaMKIIα, and the abundance of CaMKIIβ in the brain, suggest important and unique functions for CaMKIIβ, which largely remain undefined.

We generated a CaMKIIβ knockout (KO) mouse by introducing loxP sites flanking exons 7–8. The CaMKIIβ floxed mice were crossed with a CMV-Cre mouse to generate a global KO of CaMKIIβ. Using a battery of behavioral tests, we found that the CaMKIIβ KO mice had motor impairments, including ataxia, as previously reported in an independently generated KO mouse [10]. We also report here that CaMKIIβ KO mice exhibit an altered body mass composition, a reduction in anxiety-related behavior, and cognitive deficits.

## Materials And Methods

### Mice

**Figure 1:**
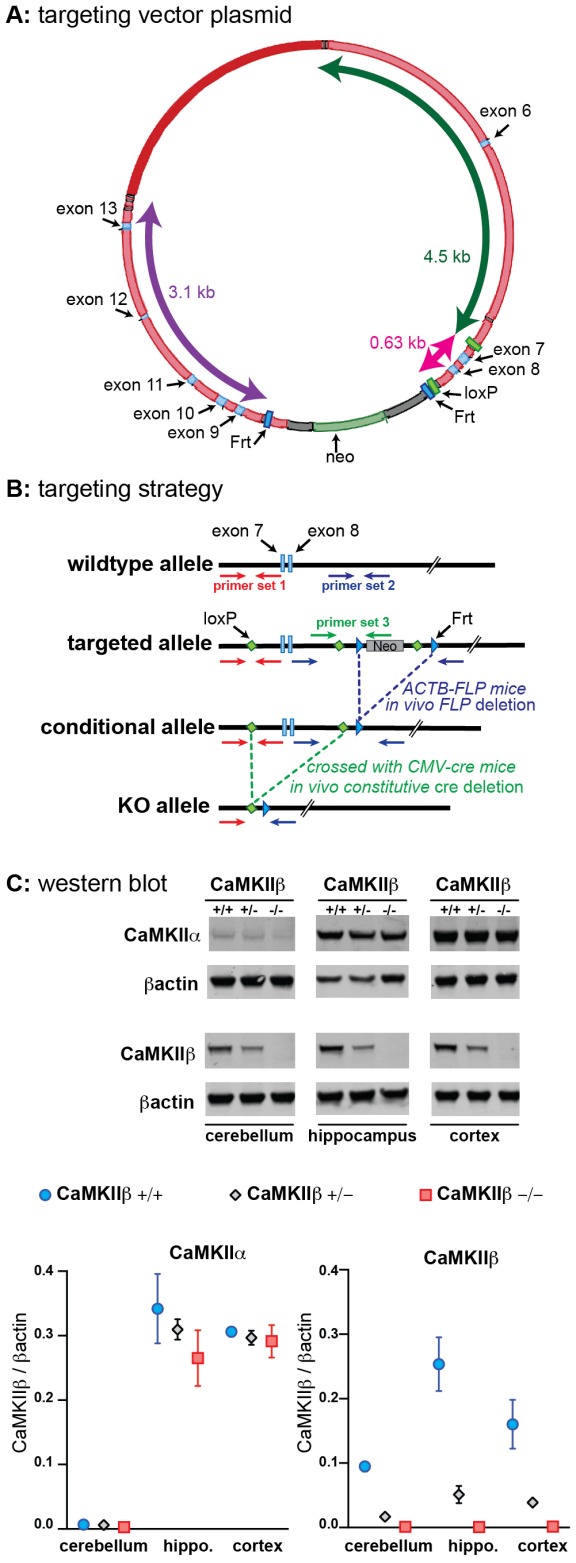
Development and characterization of CaMKIIβ KO mice. (**A**) Diagram of the targeting vector plasmid used to construct the CaMKIIβ KO. (**B**) Representations of the CaMKIIβ wildtype genomic locus, the targeted selection allele, the conditional allele, and the KO allele are shown. Location of PCR primer sets used to detect the different alleles is indicated by the horizontal arrows. (**C**) Representative western blots show the lack of CaMKIIβ protein in the KO mice with no compensatory changes in CaMKIIα levels. Data are presented as means ±SEM. n = 3–4 mice per genotype, for each brain region.

The Institutional Animal Care and Use Committees of the University of Kentucky and Northwestern University approved the use of animals in this study, which were conducted in accordance with the principles of animal care and experimentation in the Guide For the Care and Use of Laboratory Animals. The CaMKIIβ KO mouse line was generated at the Institut Clinique de la Souris – Mouse Clinical Institute (ICS-MCI; Illkirch, France; http://www.ics-mci.fr/). The targeting vector plasmid was created in a propietary ICS-MCI vector containing a Frt Neomycin resistance cassette (**Figure 1A**). The plasmid consisted of a 4.5 kb fragment encompassing exon 6, a 0.63 kb fragment encompassing exons 7–8 and flanked by loxP sites, and a 3.1 kb fragment encompassing exons 9–13. The linearized construct was electroporated in 129S2/SvPas mouse embryonic stem (ES) cells. After selection, targeted clones were identified by PCR using external primers and further confirmed by Southern blot with 5′ and 3′ external probes. Positive ES clones were injected into C57BL/6 blastocysts, and male chimeras derived gave germline transmission. Removal of the Neomycin cassette in the targeted allele was accomplished by crossing the mice with Flp transgenic mice, to generate mice with the conditional allele. Finally, the global KO mice were obtained by crossing the mice with CMV-Cre transgenic mice to generate the KO allele (**Figure 1B**).

PCR analysis was used to detect the presence of the four different alleles. Briefly, mouse tail tissue samples were digested in directPCR lysis buffer with 100 µg of Proteinase K (Viagen Biotech, Los Angeles, CA) at 56°C overnight. After digestion, 1 µl of supernatant containing mouse DNA, primers, and PCR master mix with AmpliTaq Gold DNA polymerase (Life Technologies, Grand Island, NY) was used for the PCR reaction, which was run according to manufacturer recommendations. The PCR products were analyzed by electrophoresis in a 2% agarose gel. Three primer sets were used to detect the four different alleles shown in **Figure 1B**. Primer set 1 (forward: 5′- CCAAGTTCAAACTGAGAGCTAACTTCC -3′; reverse: 5′- CCAGAAGCAGGTTCTCAGGCTACAG –3′) produces a 222 bp product from the WT allele, and a 271 bp product when the loxP site was present in the targeted and conditional allele. Primer set 2 (forward: 5′- GTATGTGTGTCATCCCTGCCTCTGG –3′; reverse: 5′- AACGGCCTCTGATCCCTGCTC –3′) produces a 282 bp product from the WT allele, a 392 bp product in mice with the conditional allele, and no product in mice with the targeted allele or KO allele. Primer set 3 (forward: 5′- CTACATTTACCAATGTGCCCCAGGC –3′; reverse: 5′- CATCTGCACGAGACTAGTGAGACG –3′) was used to detect a 592 bp product only present in the targeted allele. The KO allele produced only one 346 bp product using primer set 1 (forward) and set 2 (reverse).

Heterozygous mice were backcrossed to C57BL/6J mice (Harlan) for >10 generations. Colonies of mice were established at Northwestern University and University of Kentucky, and mice were bred and maintained as heterozygotes. Genotyping for colony maintenance was done by PCR methods or by Transnetyx, Inc (Cordova, TN). Mice were housed in temperature controlled, pathogen-free facilities with a 10:14 h light:dark cycle. Food and water were provided ad libitum.

### Body weight, and lean and fat mass determination

Body weight of WT (CaMKIIβ^+/+^) and KO (CaMKIIβ^−/−^) littermates was recorded at birth, as well as every 3–4 days from postnatal day (PND) 21 to day 60, and then weekly until PND 120. The body weight was expressed as the percent of CaMKIIβ^+/+^ littermates at that PND. Whole body lean and fat mass in 12-week old mice were quantified with a mouse-specific echo magnetic resonance imaging system (Echo MRI-500 instrument; Echo Medical System, Houston, TX). Prior to analysis, the system was calibrated using a known standard provided by Echo Medical System. Mice were restrained in a clear plastic cylinder, for ∼3 minutes while the plastic cylinder was in the scanner. The quantitative magnetic resonance spectroscopy method provided calculations of total adipose (fat) mass and apparent skeletal muscle (lean) mass [11].

### Metabolic assessment, food intake and activity analysis

The LabMaster system (TSE Systems, Chesterfield, MO) was used to measure the circadian pattern of locomotor activity and ingestion behavior as well as indirect calorimetry. Mice were placed in mock (non-recording) LabMaster cages for 1 week prior to testing to acclimate to the novel environment. After the acclimation period, the mice were transferred to recording cages at 8am. Data are recorded starting at 6am the following day, for 3 consecutive days. Animals received water and food ad libitum and remained undisturbed by the investigator during observation. The LabMaster system consists of a combination of feeding and drinking sensors, a photobeam-based activity monitoring system, and a calorimetry system to determine O_2_ consumption, CO_2_ production, and respiratory exchange ratio (volume CO_2_/ volume O_2_).

### Grip Strength

A digital force-gauging apparatus (Animal Grip Strength System, San Diego Instruments, San Diego, CA) was used to measure grip strength. The mouse was held by the nape of the neck and by the base of the tail. The forelimbs or hindlimbs were placed on the tension bar pad, and the mouse was pulled back gently until it released its hold on the grip pad. Resistance was automatically calculated in real time with the digital force-gauging apparatus, and the maximal resistance achieved by each mouse was reported as the final grip strength.

### Rotor-Rod

The mice were placed on a rotating rod (3.18 cm diameter) in lanes 11.5 cm wide to maintain the animal in the same direction while the bar is rotating (ROTOR-ROD System, San Diego Instruments). The bar is 46 cm from the floor of the apparatus, and the bar's speed of rotation was gradually and linearly increased from 0 rpm to 40 rpm across the 5-minute trial. Both the latencies (s) and the distance (cm) at which the mice fell off the bar were recorded automatically using photobeam break technology.

### Beam Walk

Mice were trained to walk along an 80 cm long and 3 cm wide beam elevated 30 cm above the bench by metal supports to reach an enclosed goal box. Following training, mice were placed on the beam at one end and allowed to traverse the beam to reach the goal box. This was repeated with beams of decreasing width (3 cm, 2 cm, and 1 cm). Foot slips were scored when one or both hindlimbs slipped from the beam.

### Open Field Activity

Mice were placed in a multi-unit open field maze (San Diego Instruments) with field chamber (50 cm long ×50 cm wide) for a 30 min trial. The field chamber was digitally divided into 25 quadrants of equal size using EthoVision XT 8.0 video tracking software (Noldus Information Technology, Leesburg, VA). The nine central quadrants are referred to as the center zone, and the 16 peripheral quadrants are referred to as the peripheral zone. EthoVision software recorded the video and scored automatically for distance traveled (cm), velocity (cm/s), and time spent in the center zone vs. the peripheral zone. Distance traveled and velocity were used as measures of ambulatory movement; the amount of time spent in the center zone vs. the peripheral zone was used as a measure of anxiety levels due to the rodent's natural thigmotaxis behavior when frightened [12].

### Running wheel

Running wheel behavior was assessed using mouse activity wheel chambers (Lafayette Instruments, Lafayette, IN) with dimensions of 35.3 cm×23.5 cm×20 cm for length, width, and height, respectively. The running wheel component consisted of a metallic wheel of 12.7 cm diameter ×5.72 cm width with 38 evenly spaced rungs for the mice to run on. Total distance and velocity run each hour by each animal was automatically recorded using Lafayette computerized counting software. The mice were given a 4 h acclimation period, before data was recorded for 12 h, from 6p to 6a.

### Elevated Plus Maze

The elevated plus maze consists of four arms (two enclosed arms and two open arms) elevated 100 cm above the floor. Anxiety-related behavior was defined as time spent in the open arms (perceived unsafe arms) of the maze, compared to the time in the closed arms (perceived safe arm) of the maze over the 5 min test session. Each mouse was placed in the center of the maze and the amount of time spent in each arm was recorded automatically by EthoVision XT 8.0 video tracking software (Noldus Information Technology).

### Visual cliff test

Visual acuity was assessed using the visual cliff test as previously described [13]. The visual cliff apparatus consisted of a chamber with dimensions of 40.64 cm×50.8 cm, bisected by a central 2.54 cm wide flat strip (ridge) raised 3.81 cm above the glass floor of the chamber. On one side of the ridge (negative side) was a 60.96 cm high cliff beneath the glass floor. On the other side (safe side) of the ridge was the floor immediately beneath the glass. All floor surfaces were covered by 2.54 cm×2.54 cm black and white checkered pattern tiles. At the beginning of each 60 s trial, a mouse was started on the ridge and the side of the chamber that the mouse decided to step down on was recorded. A score of 80%–90% for the safe side of the chamber indicates normal visual acuity across multiple strains of mice [13].

### Novel Object Recognition (NOR)

NOR behavior was assessed as previously described [14]. Briefly, the test apparatus consisted of an open field box measuring 39.37 cm×78.74 cm in diameter. On day 1, the animal was allowed to explore the open field box for a 15 min time period to habituate the mice to the novel location. Testing began on the following day. Mice were first presented with two identical objects for 10 min (A/A training session). After a four-hour delay where the mice were returned to their home cages, the mice were presented with one familiar and one novel object for 10 min (A/B test session). The objects were made of hard plastic and had previously been counterbalanced to control for any object preference bias. The total amount of time spent with each object was recorded and scored using the EthoVision XT 8.0 video tracking software. Time spent was operationally defined as occurring when an animal directed its nose to the object at a distance of less than 2.0 cm and/or by the animal touching the object with its nose or mouth. Data are presented as the D2 discrimination index. The D2 index is a common measure of discrimination between novel and familiar objects. It is considered one of the most reliable measures of discrimination because it corrects for total exploratory activity of each animal [15]. The D2 index is calculated for an A/B session by examining the difference in time spent exploring the novel and familiar objects divided by the total exploration time for both objects (D2 =  (novel - familiar) / (novel + familiar)). In this way, the D2 index corrects for total exploratory behavior of each mouse.

### Nesting behavior

Nesting behavior was assessed as previously described [16]. Briefly, as part of standard enrichment, mice receive a nestlet, a 5 cm square of pressed cotton, which they use to make a nest. To perform the test, we moved each mouse from group housing to single housing in a separate clean cage, without a nestlet. After 3 hrs without a nestlet, one nestlet was added at 17:00 to each cage, 1 hr prior to when the room lights turn off. At approximately 16 hrs (09:00) after the nestlet was added to the cage, the nest quality was scored by 2 observers blind to the experimental conditions. The five- point scale used for scoring the nest follows:

1. Greater than 90% of the nestlet intact.
2. 50–90% of the nestlet intact.
3. 10–50% of nestlet intact but no identifiable nest site.
4. Less than 10% of nestlet intact. A nest is identifiable but flat.
5. An identifiable nest with walls higher than mouse body, with less than 10% of nestlet intact.

### Euthanasia and brain tissue harvesting

Mice were euthanized with an overdose of sodium pentobarbital, and transcardially perfused with ice-cold phosphate buffered saline (PBS) for 5 min. The brains were rapidly removed and bisected along the sagittal plane. One hemisphere was further dissected and flash frozen in liquid nitrogen and stored at −80°C for subsequent biochemical evaluation. The other hemisphere was immersion fixed in 4% paraformaldehyde overnight, prior to cryoprotection in a 30% sucrose/PBS solution.

### Immunohistochemistry (IHC)

As previously described [17], sagittal sections (30 µm) were made using a sliding microtome with a freezing stage. Staining procedures were conducted on free-floating sections. The primary antibody, mouse anti-CaMKIIβ (1∶10,000, LifeSpan Biosciences, Seattle, WA, Cat no. LS-C21191), and goat biotinylated secondary antibody were diluted in 3% normal goat serum (NGS: LAMPIRE Biological Laboratories, Pipersville, PA) with 0.2% Triton X-100. Endogenous peroxidase activity was quenched with 3% H_2_O_2_ in methanol, prior to tissue blocking in 10% normal goat serum with 0.2% Triton X-100. Signal was amplified in avidin-biotin substrate (ABC kit, Vector Laboratories, Burlingame, CA). All sections were developed in 0.5 mg/ml 3,3-diaminobenzidine tetrahydrochloride solution (Sigma-Aldrich, St. Louis, MO, Cat no. D5637). The tissue sections were dehydrated through gradients of ethyl alcohol, and finally xylene. The sections were then coverslipped with Permount Mounting Medium (Fisher Scientific, Pittsburgh, PA). The Aperio ScanScope XT digital slidescanner (Aperio, Vista, CA) was used to image the entire stained slide at 20× magnification to create a single high-resolution digital image. Digital images were analyzed as described [17].

### Western immunoblot analysis

As previously described [18], tissue was homogenized using an Omni TH homogenizer, in a (1∶10 w/v) detergent- containing lysis buffer (tissue protein extraction reagent with Halt protease and phosphatase inhibitor cocktail: Thermo Scientific, Waltham, MA). Extracts were centrifuged at 12,000×g for 20 min at 4°C in a Beckman Microfuge 18, and supernatants collected for measurements. Western blots were run using 10 µg of the detergent-solubilized fraction prepared in SDS loading buffer (LI-COR Biosciences, Lincoln, NE). The sample was separated on a 10% NuPAGE Novex Bis-Tris Midi Gel (Life technologies). Proteins were transferred to nitrocellulose membrane using a dry blotting system (iBlot Life Technologies). Blots were probed using reagents and manufacturer recommendations for Odyssey Infrared imaging system (LI-COR Biosciences). Blots were probed for the following primary antibodies: mouse anti-CaMKIIα (1∶10,000, Abcam, Cambridge, United Kingdom, Cat no. ab2725); mouse anti-CaMKIIβ (1∶10,000, LifeSpan Biosciences, Cat no. LS-C21191). Mouse anti-β-actin (1∶10,000, Cell Signaling, Danvers, MA, Cat no. 37005) was used as a loading control. Blots were visualized and analyzed on the Odyssey Infrared imaging system (LI-COR Biosciences).

### Statistics

Statistical analysis was conducted using GraphPad prism software V.6 (GraphPad Software, La Jolla, CA). Groups of two were compared by unpaired t-Test. One-way ANOVA followed by Tukey's multiple comparisons test was used for comparisons among three or more groups, unless otherwise indicated. Statistical significance was defined as p<0.05. Values are expressed as mean ±SEM.

## Results

### Generation of CaMKIIβ KO mice

**Figure 2:**
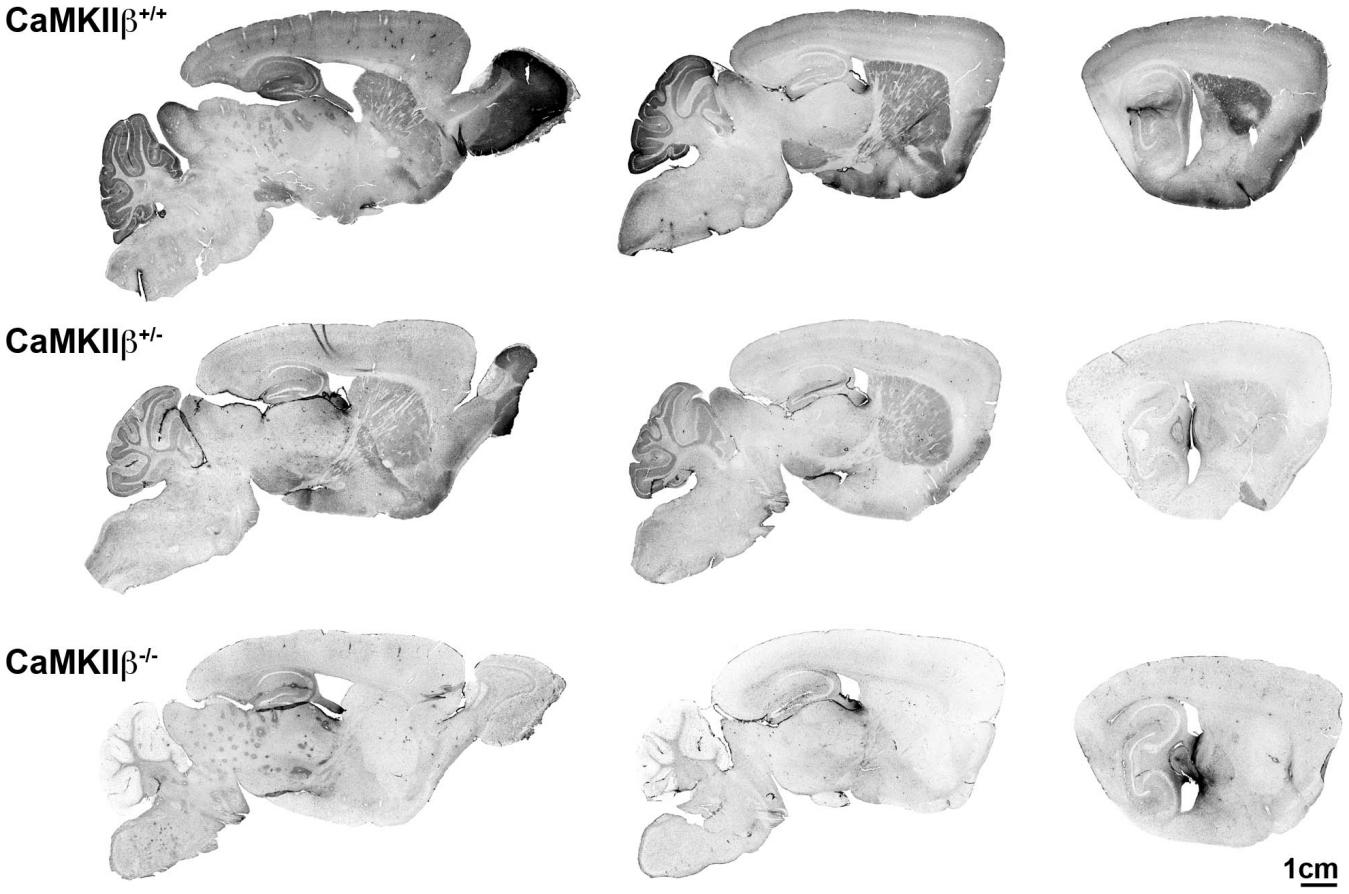
CaMKIIβ immunohistochemistry. Representative images of the CaMKIIβ IHC staining for one mouse from each genotype is shown at approximately 1 mm lateral intervals from midline. In the WT (+/+) mouse, CaMKIIβ staining is highest in the olfactory bulb, but is also apparent in the cerebellum, cortex, hippocampus, striatum, and substantia nigra. The heterozygous mice show an intermediate level of staining. The KO mice show a lack of CaMKIIβ staining throughout the brain.

As discussed in Methods, the CaMKIIβ KO mouse line was generated at the MCI/ICS in Illkirch, France. The design strategy is shown in **Figure 1A, B**. Briefly, a targeted selection allele was created by inserting loxP sites surrounding exons 7–8, and incorporating a Frt neomycin resistance cassette downstream. *In vivo* FLP deletion results in the conditional allele. CaMKIIβ floxed mice were crossed with a CMV-Cre mouse to generate the global KO. Matings between heterozygotes generated wild-type (+/+), heterozygotes (+/−), and CaMKIIβ KO (−/−) mice at a ratio of 30%, 54%, and 16% respectively for 293 mice born over a two year period. To confirm the deletion of CaMKIIβ, homogenates from the cerebellum, hippocampus and cortex, brain regions with reported high levels of CaMKIIβ, were prepared and analyzed by western blot. The KO mice showed no detectable CaMKIIβ. Importantly, no compensatory increase or decrease in the levels of CaMKIIα was found in the KO mice (**Figure 1C**). IHC evaluation of CaMKIIβ showed abundant staining in the olfactory bulb, hippocampus, cortex, striatum, substantia nigra, and cerebellum in the WT mice. A decrease of CaMKIIβ staining was seen in the heterozygotes, and a total lack of staining was seen in the CaMKIIβ KO mice (**Figure 2**).

### CaMKIIβ KO mice show a developmental delay in body weight

**Figure 3:**
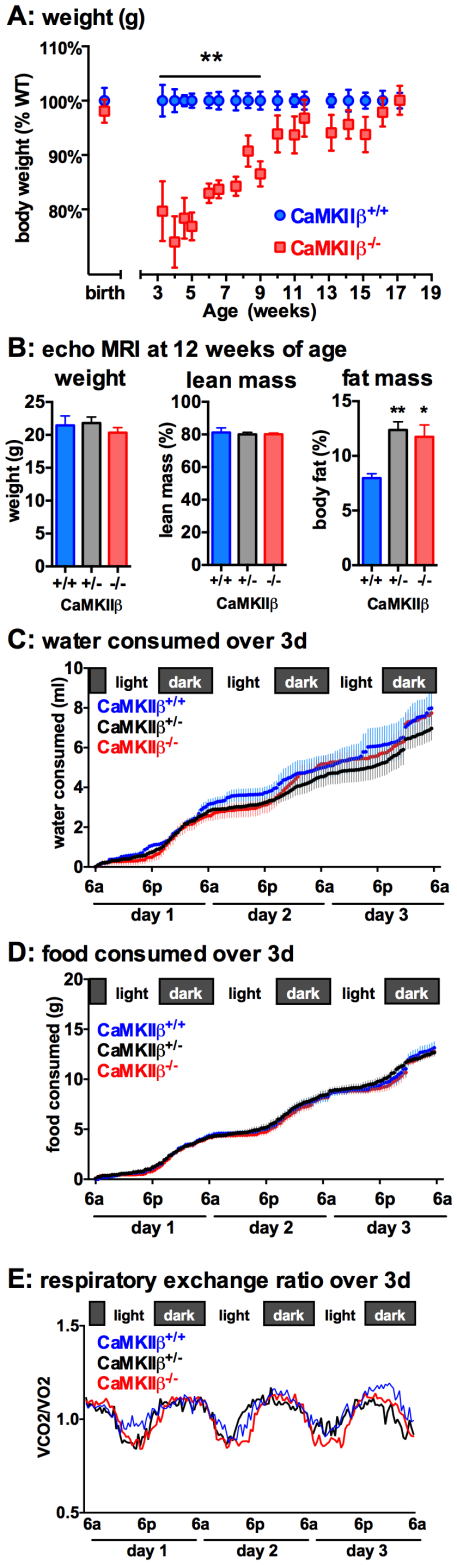
CaMKIIβ KO mice have a developmental delay in body weight. (**A**) CaMKIIβ^+/+^ and CaMKIIβ^−/−^ mice were weighed at birth, or starting at weaning (3 weeks old) until 4 months of age. n = 5–13/time point. (**B**) Echo MRI was used to determine lean/fat mass in 12 week old mice. No genotype effect was seen in the amount of water (**C**) or in the amount of food (**D**) consumed, or in respiratory exchange ratio (**E**). *p<0.05, **p<0.01. Data are presented as means ±SEM. n = 4–6 per group in panels B–E.

At birth, no effect of genotype was seen in body weight (+/+1.19 g±0.03 g n = 14; +/− 1.2 g±0.02 g n = 26; −/− 1.17 g±0.3 g n = 6). Gross histological examination of H&E stained brain tissue from 3–4 week old mice showed no abnormalities in the heart, lung, liver, spleen, or brain. However, at weaning (3–4 weeks old), CaMKIIβ KO mice were found to be smaller than their WT littermates. Body weight was measured at weaning until 4 months of age. As shown in **Figure 3A** **,** CaMKIIβ KO mice had significantly lower body weight compared to WT littermates (p<0.001) from week ∼4 to ∼9. By week 10, the differences in body weight were no longer significant.

### Body composition is altered in CaMKIIβ KO mice

To determine if the delayed developmental weight gain was altering the composition of lean mass to fat mass, the body mass composition of the mice was determined by quantitative magnetic resonance spectroscopy [11]. At 12 weeks of age, the CaMKIIβ KO mice showed no significant differences in total weight and lean mass compared to WT littermates. (**Figure 3B**). However, both CaMKIIβ heterozygotes (p = 0.0094) and CaMKIIβ KO mice (p = 0.0291) had increased percentage of fat mass compared to WT littermates (**Figure 3B**).

### Food consumption was not regulated by CaMKIIβ

An automated system with highly sensitive feeding and drinking sensors was used to determine if alterations in food or water intake could account for the increased fat mass found in the CaMKIIβ^+/−^ and CaMKIIβ KO mice. Mice were single housed in the LabMaster (TSE Systems) behavioral and metabolic monitoring cages for one week of acclimation prior to testing. During the first two days of the one week acclimation period, we observed that the CaMKIIβ^+/−^ and CaMKIIβ^−/−^ mice were not drinking water. The LabMaster (TSE Systems) metabolic monitoring cages use a hanging water bottle with a lever dispenser, instead of the more standard ball dispenser. The lever dispenser required the mice to press on the lever for water droplet to be released. In contrast, water droplet in a ball dispenser is always present. While observing the behavior of the CaMKIIβ KO mice, we found that KO mice tried to drink from the LabMaster water bottle, but could not coordinate their movement to move the lever at the tip of the water bottle spout to release a water droplet. The WT mice had no difficulties learning how to drink from the novel water bottle. During the acclimation period, experimenters primed the water bottles so that a water droplet was present at the tip of the water bottle spout. After three days of priming the water bottles, all the mice learned how to drink from the novel bottles. During the subsequent 3-day test phase (**Figure 3C**), no significant genotype effect was seen in the amount of water consumed or in the amount of food consumed (**Figure 3D** **).** In addition, no effect by genotype was found in the respiratory exchange ratio (**Figure 3E**).

Subsequent to this assay, we noticed that the home cage automatic in-cage water dispenser (Allentown) used a similar lever-activated water spout as the LabMaster water bottle. Previously, we found an increased premature mortality in the CaMKIIβ KO mice, particularly when they were individually housed. Once we started to add a water bottle with a ball type dispensing water spout, the premature mortality observed in the CaMKIIβ KO mice was no longer seen.

### CaMKIIβ KO mice show impairments in motor coordination and balance

**Figure 4:**
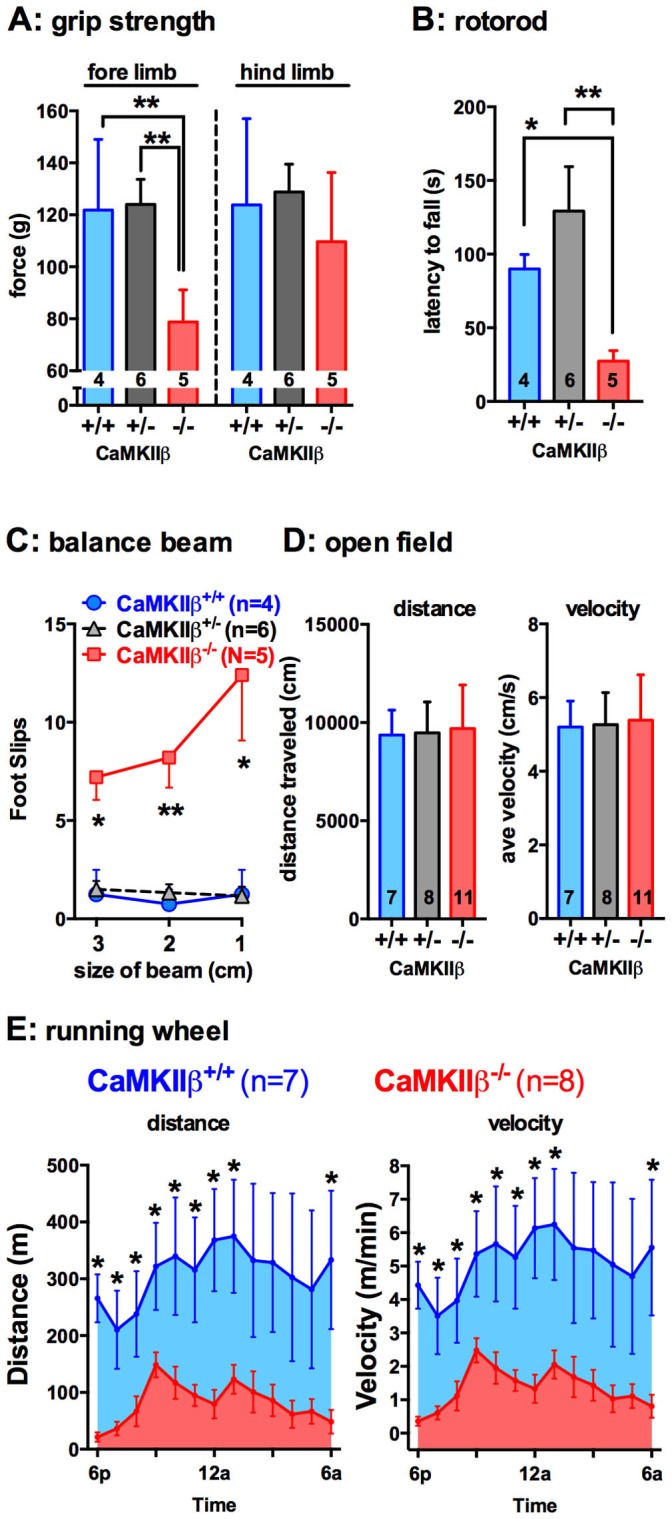
CaMKIIβ KO have impaired motor function. (**A**) CaMKIIβ KO mice show impaired forelimb grip strength but not hindlimb grip strength. The CaMKIIβ were impaired on the rotorod (**B**) and on the balance beam (**C**). No difference in spontaneous movement was seen in the open field task (**D**), but spontanous activity in the running wheel was decreased (**E**). *p<0.05, **p<0.01. Data are presented as means ±SEM. Number of mice per group is indicated on each graph.

As previously reported in an independently generated line of CaMKIIβ KO mice [10], a forelimb ataxia was also observed in our CaMKIIβ KO mice. To further assess the motor deficits caused by loss of CaMKIIβ, adult mice (3–4 months old) were tested in five motor function assays, including: grip strength (**Figure 4A**); rotorod (**Figure 4B**); balance beam (**Figure 4C**); open field (**Figure 4D**); and voluntary running wheel (**Figure 4E**). CaMKIIβ KO mice were found to release forelimb grip at significantly less tension force than the WT (p = 0.0019) or heterozygote (p = 0.0058) littermates, whereas no difference was seen in hindlimb grip strength (**Figure 4A** **)**. A significant impairment was also seen in the rotorod task. The CaMKIIβ KO mice fell after ∼30 seconds on the rotorod, compared to significantly longer latency to fall times of 1.5 to 2 minutes for the WT (p = 0.0314) or heterozygote (p = 0.0056) littermates (**Figure 4B**). The mice were subsequently tested on the balance beam task, using 3 beams with increasing difficulty (3 cm, 2 cm, and 1 cm width). The CaMKIIβ KO mice had significantly more foot slips on all 3 beam widths compared to the WT or heterozygote littermates (**Figure 4C**). No difference was found in the open field task in total distance traveled or in the velocity of movement (**Figure 4D**). However, in the voluntary running wheel the CaMKIIβ KO mice were found to run significantly less total time than the WT mice and at a significantly slower velocity (**Figure 4E**).

### A reduction in anxiety-related behavior is seen in CaMKIIβ KO mice

**Figure 5:**
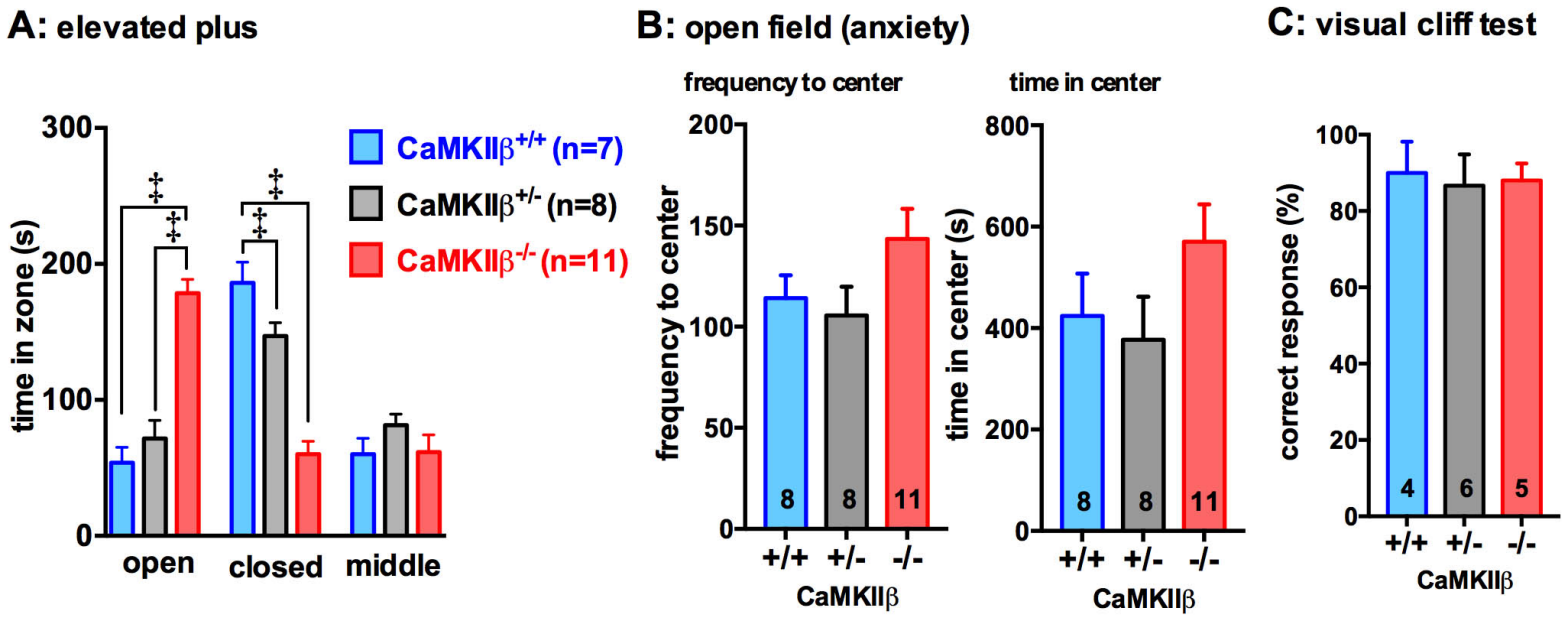
CaMKIIβ KO show reduced anxiety-related behaviors. (**A**) In the elevated plus maze task, the CaMKIIβ^+/−^ and CaMKIIβ^−/−^ mice spent more time in the open arms and less time in the closed arms compared to the CaMKIIβ^+/+^ mice. (**B**) The KO mice also spent more time in the center of the open field. (**C**) Using the visual cliff test, the CaMKIIβ KO mice showed no deficit in vision (depth perception). Data are presented as means ±SEM. ^‡^p<0.0001 Number of mice per group is indicated on each graph.

To assess if anxiety-related behaviors were associated with deficiency of CaMKIIβ, two behavioral tests were run: the elevated plus maze task (**Figure 5A**) and the time spent in the center zone versus peripheral zone of the open field arena (**Figure 5B**). In the elevated plus maze, the CaMKIIβ KO mice spent significantly more time in the open arm, and less time in the closed arm of the elevated plus maze compared to WT (open p<0.0001; closed p<0.0001) or heterozygote (open p<0.0001; closed p<0.0001) littermates (**Figure 5A**). Similarly, in the open field test, the CaMKIIβ KO mice entered the center of the field more often and spent more time in the center of the field than the WT or heterozygote mice, although these differences did not reach significance (**Figure 5B**). To determine if deficiency in CaMKIIβ could impair vision, thus potentially explaining the differences seen in the elevated plus mice, we used the visual cliff test. The visual cliff test is a useful task in C57BL/6 mice to determine depth perception [13]. We found that greater than 80% of the time the mice, regardless of genotype, chose to step off the beam on to the shallow checkered (safe) side of the box. These results indicate that the mice had the visual ability to perceive depth. Therefore, the anti-anxiety phenotype of the CaMKII KO mouse in the elevated plus maze is unlikely to be explained by an inability to visually determine the difference between the open and closed arms of the maze.

### Novel object recognition is impaired in CaMKIIβ KO mice

**Figure 6:**
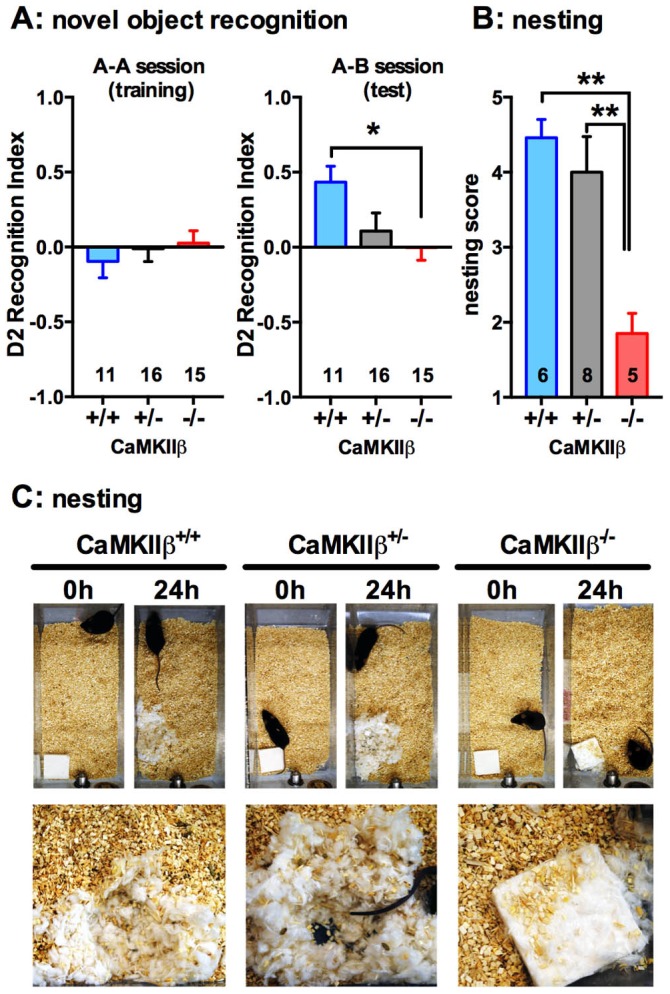
CaMKIIβ KO mice have impaired cognitive function and nesting behavior. (**A**) None of the mice show a preference for two identical objects presented in the NOR training session. In the subsequent test session done after a 4 hour delay, the WT mice showed a preference for the novel object as indicated by increased D2 index, but the KO mice showed no preference for either object. (**B**) CaMKIIβ KO mice made a significantly lower quality nest compared to their littermates. (**C**) Representative photographs of the nests. Photographs on the bottom row are higher-power views of the nests at 24 h. *p<0.05, **p<0.01. Data are presented as means ±SEM. Number of mice per group is indicated on each graph.

Spatial learning in rodents is often tested using a water maze task; however, the motor impairments in the CaMKIIβ KO mice precluded the use of this common cognitive task. As an alternative test to measure cognitive function, we used the novel object recognition (NOR) task. This task of recognition memory utilizes the natural tendency of mice to spend more time exploring a novel object compared with an object they are familiar with, in order to satisfy their innate curiosity / exploratory instinct. No difference was seen between genotypes in the time spent exploring two identical objects in the A–A training session. In the NOR test performed 4 hours after the training session, the WT mice showed a preference for exploring the novel object, as expected. However, the CaMKIIβ KO mice showed no preference for the novel object. The CaMKIIβ^+/−^ mice showed an intermediate response, demonstrating reduced preference for the novel object compared to the WT littermates, although by an unpaired t-test this effect did not quite reach significance (p = 0.064; +/+ vs +/−) (**Figure 6A**).

### CaMKIIβ KO mice fail to build nests

Nesting is a natural behavior of mice associated with ‘activity of daily living’. With the exception of pregnancy and lactation, nesting is largely related to thermoregulation, but also with exploration and camouflage [19]–[21]. The WT mice and CaMKIIβ^+/−^ mice built high quality nests with walls higher than the body of the mouse. In contrast, the CaMKIIβ KO mice left the nestlet largely untouched (**Figure 6B and 6C**).

## Discussion

We report here the development and comprehensive behavioral phenotyping of mice deficient in CaMKIIβ. The CaMKIIβ KO appears to have no major effect on CaMKIIα expression, thereby allowing a complementary and independent analysis of the phenotypic contributions of CaMKIIβ vs CaMKIIα. The behavioral characterization reveals discrete effects of the KO, providing a foundation for future investigations. For example, this is the first description of a cre/loxP system for modulation of CaMKIIβ knockout, thereby allowing a more precise analysis of CaMKIIβ function specific cell types, tissues or development stages in future research.

The results presented here are in agreement with data from an independently generated CaMKIIβ KO mouse [9], [10], in that our CaMKIIβ KO mouse also shows a lack of motor coordination, and exhibits cognitive impairments. In addition, we show here that CaMKIIβ KO mice have decreased levels of anxiety-related behavior and a developmental delay in body weight gain. This study extends previous work on the CaMKIIβ isoform, clearly demonstrating the fundamental importance of CaMKIIβ isoform in many aspects of the mouse behavior. Global deletion of CaMKIIβ resulted in ataxia, which largely affected forelimb coordination and strength, as observed by watching the movement of the mice, and measured experimentally by the grip strength analysis. Body mass composition indicates that this effect was not due to less muscle mass; therefore, the grip strength deficit appears to be nervous system dependent. Interestingly, the motor impairments required a total loss of the CaMKIIβ, as the CaMKIIβ^+/−^ mice were found to have no motor impairments. CaMKIIβ is known to have critical roles in cerebellar Purkinje cell synaptic function [10]; thus, it would be logical to assume that the ataxia is cerebellar in origin.

In addition to the high expression of CaMKIIβ in the cerebellum, our results also demonstrate high levels of CaMKIIβ in the basal ganglia circuit (i.e. striatum, substantia nigra), in agreement with previous studies showing CaMKIIβ expression in tyrosine hydroxylase positive neurons in the substantia nigra [22] and in striatal medium spiny neurons [23]. CaMKIIβ has also been reported [24] to have important functions in oligodendrocyte maturation and in myelination. Therefore, motor impairments in our CaMKIIβ KO mice could be a result of myelin deficits in the spinal cord, basal ganglia dysfunction, or cerebellar dysfunction. Future studies are needed to tease apart the function of CaMKIIβ in these different systems.

The motor impairments in the CaMKIIβ mice may contribute to the observed decrease in body weight compared to the WT littermates at weaning. No difference was seen in body weight at birth, and therefore the decreased body weight at weaning likely reflects a failure to compete with littermates for milk. However, even after the CaMKIIβ KO mice no longer weighed less than the WT littermates, there was still an alteration in body mass composition, with an increased percentage of fat mass that appears highly sensitive to loss of CaMKIIβ. Some evidence indicates that CaMKIIβ may have a function in regulating growth hormone release from the pituitary [25], but more work is necessary to determine the mechanisms behind the changes in body size / composition observed in the CaMKIIβ KO mice.

An interesting finding during the characterization was reduction in anxiety-related behavior. While we do not know the mechanism of this reduced anxiety-related behavior, it appears to be gene dose dependent in that the CaMKIIβ heterozygotes exhibited a behavioral phenotype intermediate to that of the WT and KO mice. In addition, CaMKIIβ in neurons in the lateral habenula was reported [26] to be involved in affective disorders, in that depression caused elevation in CaMKIIβ in the lateral habenula, and anti-depressants decreased CaMKIIβ levels. Similarly, overexpression of CaMKIIβ in the lateral habenula can induce a depressive phenotype which can be reversed by downregulation of CaMKIIβ by RNAi [26].

Although much of the work on the role of CaMKII in hippocampal LTP has focused on the αisoform of CaMKII, a role of CaMKIIβ in LTP has also been documented. For example, the ratio of CaMKIIαto CaMKIIβ in the holoenzyme is known to affect synaptic strength [27]. However, the role of CaMKIIβ is thought to be mainly through its non-catalytic domain [9]. CaMKIIβ is also important for structure of the synapse. For example, CaMKIIβ is involved in the developmental changes in dendrite elaboration and dendrite pruning [6], . While the motor impairment excluded the use of water-maze tasks, we were able to use the NOR recognition task to show that the CaMKIIβ KO mice had impaired learning. These results are in agreement with previous studies using contextual fear conditioning to demonstrate cognitive impairments in CaMKIIβ KO mice [9].

## Conclusions

We report the first cre/loxP-based CaMKIIβ KO mouse strain. The initial characterizations here of the global CaMKIIβ KO demonstrate and extend the phenotypic findings for the motor and cognitive deficits previously identified in CaMKIIβ KO mice. The potential to fine-tune the CaMKIIβ KO in our mouse strain makes it a valuable research tool for future investigation of context-dependent functions of this important enzyme.
